# The molecular mechanisms of diapause and diapause-like reversible arrest

**DOI:** 10.1042/BST20221431

**Published:** 2023-10-06

**Authors:** Sreesankar Easwaran, Denise J. Montell

**Affiliations:** Molecular, Cellular, and Developmental Biology Department, University of California, Santa Barbara, CA 93106, U.S.A.

**Keywords:** chemotherapy, diapause, dormancy, germline stem cell, quiescence, stem cell self-renewal

## Abstract

Diapause is a protective mechanism that many organisms deploy to overcome environmental adversities. Diapause extends lifespan and fertility to enhance the reproductive success and survival of the species. Although diapause states have been known and employed for commercial purposes, for example in the silk industry, detailed molecular and cell biological studies are an exciting frontier. Understanding diapause-like protective mechanisms will shed light on pathways that steer organisms through adverse conditions. One hope is that an understanding of the mechanisms that support diapause might be leveraged to extend the lifespan and/or health span of humans as well as species threatened by climate change. In addition, recent findings suggest that cancer cells that persist after treatment mimic diapause-like states, implying that these programs may facilitate cancer cell survival from chemotherapy and cause relapse. Here, we review the molecular mechanisms underlying diapause programs in a variety of organisms, and we discuss pathways supporting diapause-like states in tumor persister cells.

## Introduction

In the wild, animals are faced with predictable adverse conditions such as seasonal changes in temperature and humidity as well as unpredictable challenges. Since most animals, unlike humans, are unable to build and maintain optimal environments, they have evolved mechanisms to adapt or escape. For example, many animals migrate long distances to avoid harsh conditions [1]. Other animals have evolved mechanisms to cope with harsh environments such as torpor, hibernation, or reptilian brumation, amongst others. Torpor and hibernation are physiological states in which animals reduce their body temperature and metabolic rate to conserve energy. Whereas torpor occurs during periods of environmental stress or food scarcity, hibernation is a seasonal state that some mammals enter during winter months. Brumation is a similar state of dormancy or reduced metabolic activity combined with lowered body temperature found in reptiles during colder months. These states share in common that they may remain in a state of sleep or rest for an extended period, lower their body temperature, and reduce the metabolic rate to conserve energy yet differ in terms of the animal groups involved, duration, frequency, and the extent of temperature reduction. Diapause is a coping strategy triggered by environmental cues such as changes in temperature, day length, or food scarcity and is employed by a variety of organisms, from invertebrates to vertebrates including mammals. During diapause, an organism essentially ‘pauses' its normal growth, development, activity, and reproduction to conserve energy and increase its chances of survival in adverse conditions.

These kinds of defensive mechanisms are common in the wild but frequently ignored when we study common model organisms in the laboratory since we tend to rear them under uniform conditions that are usually optimized for rapid growth and reproduction. Studying this developmental plasticity in response to stressors is important if we wish to understand how they relate to development under optimal conditions and uncover protective pathways that confer resilience to environmental changes.

**Figure 1.**
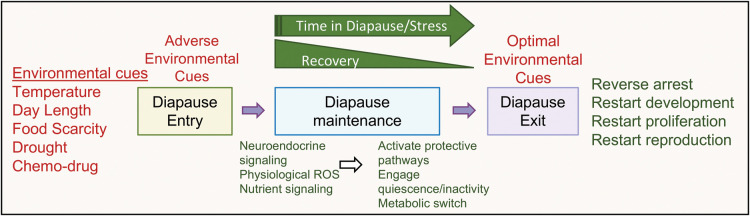
Diapause/diapause-like state to survive adverse conditions. Animals or cells sense the environmental cues or chemo-drug treatment and transform into a protective stress-resistant state to evade the harsh conditions. Once the adverse conditions reverse, they can then recover and restart the development or the proliferation. The extent of reversibility depends on the duration and intensity of the adverse conditions prevailing, and the sooner the optimal conditions arrive better the reversibility.

During diapause, organisms either arrest or delay development at different preferred lifecycle stages in response to regular and recurring periods of adverse environmental conditions (Figure 1). Development resumes once the conditions become favorable. To undergo diapause an organism must (1) sense the environmental cues, (2) enact changes to its physiology, (3) halt development, and (4) remain quiescent until conditions become favorable, then (5) sense the improving environmental condition, and finally (6) reverse the diapause process and restart normal development. Genes involved in any of these processes would be expected to modulate the ability to diapause and perpetuate the species.

Understanding diapause is fundamental to harnessing the underlying mechanisms. It is of interest to determine whether these diverse responses to environmental adversity share common features or whether there may be multiple mechanisms that can confer resilience. Furthermore, studies of recovery mechanisms will uncover whether organisms reactivate normal developmental programs or employ new ones. The mechanisms may also relate to evolution, as environmental stresses may elicit phenotypic changes that can later become fixed by regulatory mutations [2]. Studying diapause contributes to a deeper understanding of ecological adaptations, offering insights into the strategies organisms employ to survive and reproduce in challenging conditions. This knowledge can have implications for fields ranging from ecology and evolution to agriculture, conservation, and medicine. Thus, diverse animal models have emerged to study the different facets of the diapause process.

## Diapause across the animal kingdom

Diapause is a significant and widely distributed phenomenon in the insect world, and different species undergo diapause at distinct lifecycle stages (embryonic, juvenile, or adult) [3–5]. For example, while silkworm and mosquito diapause occurs in the embryonic stage, monarch butterflies and cotton bollworms diapause in the pupal stage, and the optimal stage for the fruit fly *Drosophila melanogaster* is the newly eclosed adult. In *Drosophila melanogaster*, the phenomenon was originally described as adult reproductive diapause due to arrested oogenesis and, more recently, spermatogenesis [6]. However, it has been variously described as a shallow diapause or as a cold-induced general stress response because the arrest to oogenesis occurs after rather than before the beginning of yolk deposition, similar to the response to stress like starvation [7]. However, detailed characterization of ovarian development uncovered differences between diapause and stresses including protein deprivation and predator exposure. These differences include early arrest of oogenesis and changes in the germline stem cell state that prolong longevity, more consistent with a dormancy [8]. The difference may lie in the details of the diapause induction conditions. When pharate adult or newly eclosed flies are cultured under short day length and at 10°C, diapause arrest is early and 100% penetrant. Shifting flies to diapause conditions days after eclosion and mating results in a less complete arrest and poorer survival. Thus true diapause may be limited to the newly eclosed virgin fly.

**Figure 2.**
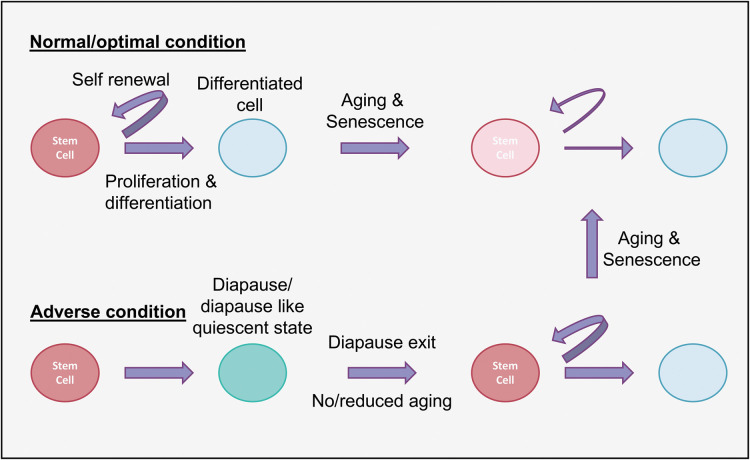
Diapause/diapause-like state protects stem cell longevity. Stem cells undertake a reversible quiescence and evade the adverse conditions to avoid senescence and decline to keep a youthful state. Post-diapause, the stem cells reverse the quiescence and resume proliferation and self-renewal.

In the nematode, *C. elegans* diapause is known as the dauer state, which occurs when first instar larvae form dauers instead of progressing to second instar larvae. Dauer formation may be the best-studied dormancy program and has been exploited for decades for insights into normal aging and possible strategies for lifespan extension [9–12]. Reduced insulin/IGF-1 signaling leads to the activation of Foxo (Forkhead box O) transcription factors to promote dauer development, demonstrating the importance of Foxo in orchestrating a switch between growth and diapause in these tiny roundworms. The discovery that the insulin signaling pathway, which is inhibited during dauer formation, promotes aging under optimal growth conditions in organisms as diverse as worms, flies, and mammals may represent the strongest evidence that studies of diapause, even in relatively simple animals, may yield general principles of resilience and longevity. The observation that diapause preserves the long-term viability of germline stem cells, presumably to ensure fertility when favorable conditions return [8,13] raises the possibility that other types of adult stem cells may employ similar mechanisms to extend their lifespans and/or health spans during diapause (Figure 2).

Recently vertebrates like killifish [14–16] have emerged as interesting embryonic diapause models [17–21]. In killifish, diapause is a remarkable survival strategy that empowers the embryos to endure and persist in habitats prone to drying up due to temperature extremes. Transcriptome analysis of this stage revealed that diapause is an active program in which polycomb complex members repress metabolism and muscle genes by maintaining H3K27me3-mediated repressive epigenetic marks on key developmental genes [15].

Close to 130 species of mammals also undergo diapause during early embryonic development in the uterus by receiving cues from the environment such as harsh seasons or lack of sufficient nutrition like during the lactation period. In mice, diapause can occur at the blastula stage if conceived during the lactating period which is characterized by delayed implantation in the uterus [22,23]. In addition to its commercial and pest control potential, diapause may have implications for human health. At the organismal level, diapause has gained attention as a potential means to extend lifespan. Since diapause extends the lifespan in many organisms, may offer clues to slowing aging. At the cellular level, it is becoming clear that cancer persister cells mimic a diapause-like state to evade cell death from chemo- and radiation therapies [24–26]. Although different organisms undergo diapause at distinct life stages and in response to a variety of cues, the decision to enter diapause and exit in most, if not all, organisms is a response to environmental change.

## Environmental sensing leading to diapause entry and exit

Diapause is typically a seasonal event and sensing the environment is fundamental for entry and exit into this program. Environmental cues such as day length and temperature are the most common diapause regulators. Day-length-sensing and tracking suggest that circadian rhythms might be involved. Consistent with that idea, the neuropeptides pigment dispersing factor (PDF) and short neuropeptide F (sNPF), which are expressed in so-called clock neurons, as well as genes controlling circadian activities like period (per), timeless (tim), eyes absent (eya), and cryptochrome (cry) affect by lowering reproductive dormancy if their function is impaired in insects [27–33]. Interestingly, similar to diapause, circadian rhythms can also modulate torpor by influencing the timing and duration of torpor [34,35].

Transient Receptor Potential (TRP) cation channels, first discovered in *Drosophila* [36,37] are well-established temperature sensors [38,39]. Temperature sensing regulates diapause through TRPA1 in silkworms and *C. elegans* [40–42]. Apart from thermosensation via sensory neurons, food scarcity and overcrowding can also induce diapause. Chemosensation plays an important role in identifying dauer pheromones while overcrowding can be sensed by touch in worms [43,44]. After sensing adverse conditions, organisms must initiate the diapause program. However, the sensors for the salient environmental cue to enter diapause are not yet known in many organisms.

## Transition to diapause via neuro-endocrine signaling

After perceiving the relevant environmental cue, hormonal and neuro-endocrine signaling rapidly signals a global change from active development to the state of dormancy or diapause. Multiple neuropeptides and hormones have been shown to regulate diapause in multiple species. For instance, the neuropeptide-like diapause hormone(DH) controls embryonic diapause in silkworms [32,40,42,45]. Integrating environmental cues such as temperature and photoperiodism can be key to successful diapause. Recent reports suggest that fruit fly pigment dispersing factor (PDF) neuropeptides, expressed in the clock neurons, are temperature- and light-sensitive and in response to diapausing conditions (short-day length and low temperature), PDF levels go down which promotes diapause ovarian arrest [30,32,34].

Juvenile hormone(JH) is a multifunctional hormone in insects that controls metamorphosis and reproduction, and it must be down-regulated for them to enter reproductive diapause [8,46,47]. Interestingly, loss or reduction in JH can also extend the fly lifespan [48]. Allatostatin C (AstC) is a neuropeptide that plays a role in the regulation of fruit fly physiology and behavior, and low temperature-mediated down-regulation of AstC activates cholinergic AstC-receptor neurons to induce ovarian arrest possibly by inhibiting JH production [49,50].

Noradrenergic signaling through norepinephrine (NE) in vertebrates or octopamine (OA) in invertebrates can regulate oocyte quiescence in response to environmental factors like absence of mates in *C. elegans* or starvation in fruit flies and zebrafish [51]. Feeding is reduced in most diapausing animals, suggesting that noradrenergic signaling in combination with nutrient signaling may play a role in the diapause process.

Another stress-resisting state common among birds and heterothermic mammals (mammals that can vary their body temperature), is torpor [52,53]. While torpor may last only a few hours in small mammals and birds such as hummingbirds, awakening from it can occur in a matter of minutes. A recent report revealed that a specific subset of glutamatergic neurons in the medial and lateral preoptic area of the hypothalamus regulates torpor in mice and inhibition of this neuronal activity prevents natural torpor process [54]. It would be interesting to determine to what extent stress resistance and response pathways overlap in torpor and diapause states.

In mammals, embryo implantation at the blastocyst stage is delayed in response to diapause. Normally, the endometrial estrogen E2 induces leukemia inhibitory factor (LIF) which initiates implantation. This signaling is down-regulated during the dormant state of the blastocyst, and E2 injection can reverse the diapause arrest [55–58]. Once initiated, diapause is maintained through the perception of additional signals like nutrient availability. Absence of E2 reduces the embryo's metabolism and activity to induce quiescence.

## Nutrient sensing and metabolic changes in diapause

Diapause can be influenced by nutrient-sensing signaling pathways. For instance, lack of food or overcrowding can initiate *C. elegans* dauer. Sensory neurons in *C. elegans* express G protein-coupled receptors (GPCRs) on their surfaces. These GPCRs are sensitive to environmental cues, including nutrients and odorants. Activation of GPCRs triggers the production of intracellular second messengers like cAMP or cGMP within the sensory neuron that relay the signal from the cell surface to downstream effector proteins in the cell. Nutrient sensing via neurons that express TAX-2/4 (α and β subunits of cGMP-gated channel) is necessary for worms to undergo quiescence as the loss of egl4, which encodes a protein kinase downstream of cGMP signaling hampers quiescence [59]. While nutrient deprivation can induce diapause, most organisms feed less during diapause that is triggered by other cues, leading to dramatic changes in nutrient signaling. Thus diapause involves sensing nutrients and in turn changes in feeding behavior, and subsequent responses to caloric restriction and changes in dietary composition [4,60–62].

The best-known nutrient signaling involved in diapause regulation is the insulin pathway, which is down-regulated at an organismal level in most diapause states across species [9,63–67]. Insulin is a key hormone involved in nutrient sensing and modulates metabolic responses to adjust energy expenditure according to nutrient levels. The relationship between Target of Rapamycin (TOR) signaling and diapause is an example of how cellular pathways originally associated with growth and metabolism can also play a role in regulating adaptive responses to environmental challenges. TOR signaling, which responds to amino acid levels, is down-regulated in some insect and mammalian diapause states [20,68–70]. Another nutrient signaling pathway that is down-regulated in diapause-like quiescence is the fatty acid oxidation (FAO) pathway — a metabolic process that generates energy. Embryonic stem cells are prompted to enter quiescence through the inhibition of FAO — by suppressing histone acetyltransferase MOF (Males absent on the first), which is a direct activator of FAO by H4K16ac of FAO pathway genes [71]. This report unveils a regulatory mechanism by which MOF sustains ESCs in an active state, providing a connection between epigenetic processes and metabolic pathways that govern cell behavior.

Animals in torpor or diapause can undergo periodic metabolic arousal while in dormancy by undergoing a metabolic switch from anaerobic to aerobic metabolism. A decreased level of reactive oxygen species (ROS) induces metabolic arousal and elevated ROS extends the duration of metabolic depression by regulating flux through the tricarboxylic acid cycle [72]. Another example of a metabolic changes in diapause is in the cotton bollworm pupae. During normal pupal development of cotton bollworms, the fat body dissociates into single cells, which is important for proper lipid metabolism to meet the energy needs of normal transition from pupal to adult stage. In diapause the dissociation fails, resulting in impaired lipid metabolism and leading to developmental arrest at pupal stage [73]. Thus nutrient signaling and metabolic switching are common and essential features of dormancy. In addition to these altered signaling events and the shift from active development to quiescence, protective mechanisms are activated in diapause to enhance survival despite extreme conditions.

## Diapause-induced protective mechanisms

During diapause, animals alter their behavior and lower activities such as feeding, movement, and reproduction. Conditions that induce diapause are generally too harsh to support rapid development, growth, and reproduction. Protective pathways must be engaged to prevent irreversible damage [19]. The best-known protective mechanisms are reduced rates of cell division or cellular quiescence. Molecular and cell biological details of protective mechanisms are also starting to emerge. The ability to preserve reproductive capacity is essential for the perpetuation of the species. In diapausing *C. elegans* most of the germline cells undergo apoptosis but a few surviving cells persist and recover when favorable conditions return [13]. In *Drosophila,* most germline stem cells enter an unusual inactive state during diapause and resurge once the environment becomes favorable [8]. Similar protective mechanisms are observed in various organisms during diapause, ranging from *C. elegans* and *Drosophila* to mammalian embryos. Growth and metabolism are down-regulated in diapause to reduce cellular activity.

In mammalian embryos, Myc or mTOR down-regulation can induce diapause in the preimplantation blastocyst stage. Micro RNAs (miRNAs) are a class of small RNA molecules that play crucial roles in post-transcriptional gene regulation. The miRNA Let-7 was initially discovered in the nematode *C. elegans*, which regulates the timing of developmental transitions. A family of related miRNAs in mice and roe deer were later found to induce diapause by down-regulating both Myc and mTOR pathways [20,74]. In both mammalian blastocysts and embryonic stem cells (ESCs), experimental reduction in Myc [75] and/or mTOR [19] reduces cell proliferation, inducing a reversible dormancy. The gene expression signatures of mTOR-depleted or Myc-depleted ESCs are similar to the gene expression patterns of diapausing blastocysts, suggesting that ESCs may enter a state of dormancy in response to starvation or experimental inhibition of mTOR or Myc and emerge again when the stress is removed [19,75,76].

An interesting unanswered question is how protective pathways are induced. One concept is that relatively low levels of induction of stress response pathways are protective whereas high levels of stress signaling can be harmful. Supporting this idea, increased but sub-pathological ROS levels that occur in diapause can induce DNA damage and DNA damage responses [8,15,77–80]. p53 is an important tumor suppressor, with roles in genome protection and cell cycle regulation in response to damage, and recent reports suggest that p53 protein levels are elevated during diapause in flies, worms, and mice, although the significance of this up-regulation is not yet clear [8,81,82].

The forkhead box family of transcription factors (FOX) is one of the key signaling nodes activated in responses to starvation or other stress conditions. Foxo expression or DAF-16 activation promotes diapause and dauer [64,83–85]. Foxo3 is up-regulated in blastocyst diapause in mice [86]. However, Foxa2 depletion in the mouse uterine gland causes embryonic diapause suggesting differential action of FOX transcription factors in different cell types such as in the uterus and blastocyst [57]. In general, FOX transcription factors can reduce transcription to slow down metabolism and limit cell divisions, thereby slowing growth and development, which allows time for DNA repair, all of which likely promote stress resistance.

The signaling pathways discussed above suggest that there are mechanisms acting to switch organisms between states: under optimal temperature, hydration, and nutrient availability, metabolism ramps up to promote growth and reproduction whereas when conditions are less favorable, organisms enter quiescent states and yet are poised to reactivate development if and when conditions improve. The first organisms to evolve such stress responses were likely unicellular, so it should not be surprising that similar mechanisms operate at the level of individual cells. For example, when cancer cells are exposed to chemotherapy, evidence is emerging that they can evade death by taking up a dormant state similar to that of diapause.

## Persister cancer cells mimic a diapause-like state for their survival

**Figure 3.**
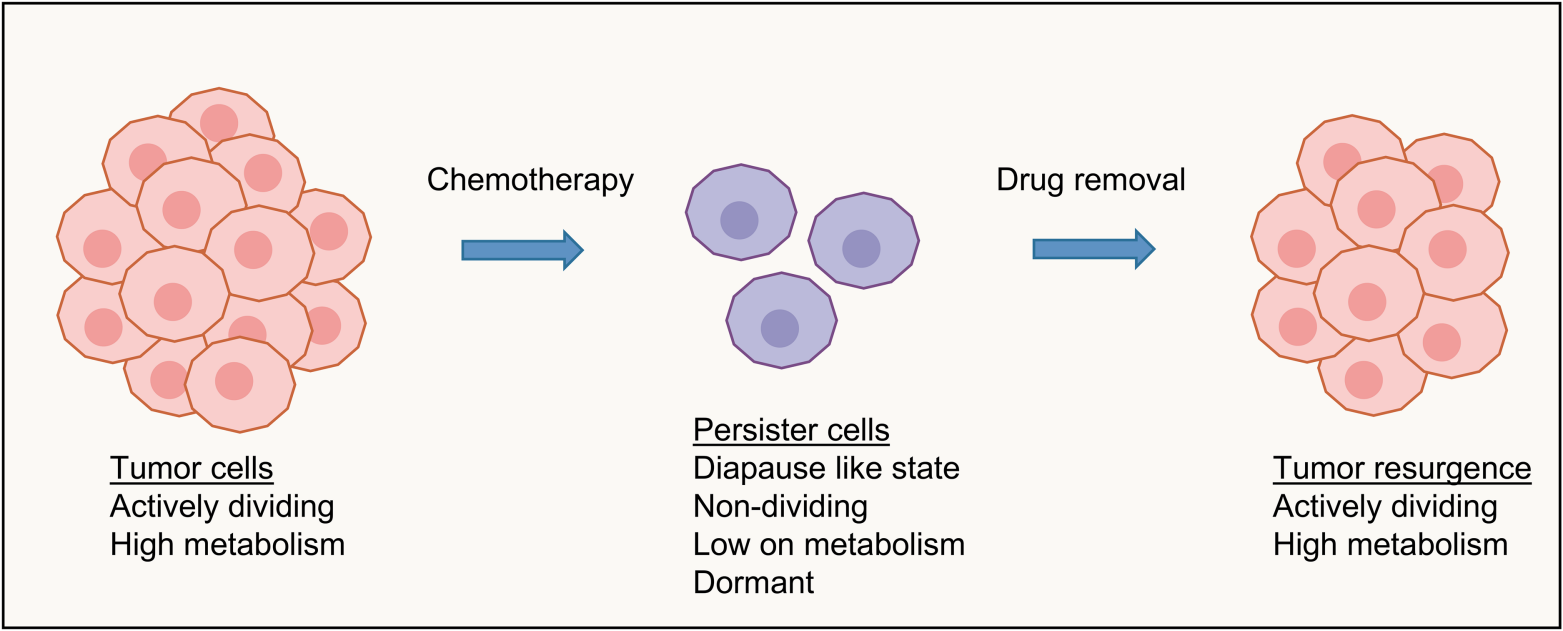
Tumor cells mimic diapause-like state to persist chemotherapy. Tumor cells take up an inactive diapause-like state to survive the chemotherapy. Once the drug is withdrawn, the persister cells can often resurge back later in life.

Adverse conditions can trigger diapause-like states not only in organisms but also in cells like embryonic stem cells (ESCs) (Figure 3). Cancer cells that survive chemotherapy drugs are the leading cause of relapse and death. Recently, it has been reported that drug-induced persister cells enter a diapause-like state thereby escaping the lethal effects of the drugs [24–26]. These reports show that persister cells have a transcriptional signature similar to that of a diapausing blastocyst, including down-regulating both Myc and mTOR pathways. This leads to a temporary quiescent state that allows survival during drug treatment. Once the drug is withdrawn, persister cells can exit quiescence and resume proliferation, leading to relapse.

Due to their high proliferation rates, cancer cells experience high ROS, which is detrimental to normal cells. In cancer, oxidative stress inhibits cell division and causes metabolic changes that can allow cells to survive, resulting in persister cells [87]. Persister cells up-regulate TRPA1, which induces Ca^2+^ influx and prevents apoptosis by overexpressing Ca^2+^-mediated anti-apoptotic signaling pathways allowing cells to resist and survive chemotherapy-induced ROS. Inhibiting TRPA1-mediated redox sensing can increase chemosensitivity and reduce persister cells [87]. Another survival mechanism identified in persister cells is the intracellular cholesterol transporter protein, NPC1L1, which can promote uptake of the antioxidant vitamin E and thereby reduce oxidative stress, and enhance persister cell survival. Blocking NPC1L1 prevents tumor survival in mice after chemotherapy [88]. Similarly, increased ROS levels are detected in developmental diapause in insects like fruit flies and cotton bollworms, although it is not yet known whether protective mechanisms are induced in response. These reports suggest similarities between developmental diapause and the diapause-like state that persister cells adopt to survive in chemotherapy.

## Conclusion

Diapause is a type of dormancy program initiated in response to harsh conditions in wild habitats that promote animal survival by halting growth and development until conditions improve. Cancer cells may coopt such dormancy pathways to persist through chemo- and radiation treatment. The molecular and cell biological details of normal and pathological dormancy programs are only beginning to be elucidated. How animals perceive environmental stresses and the role of neuroendocrine control of diapause programs are starting to emerge. Further in-depth mechanistic understanding of the protective pathways engaged during diapause and diapause-like states is needed. Manipulating and engaging the protective pathways has the potential to increase organismal resilience to climate change and in principle might be manipulated to control diseases like cancer and insect-borne diseases, extend stem cell longevity, reproductive span, healthspan, and even lifespan.

## Perspectives

- Organismal resilience to stress, with implications for evolution and climate change, can be better understood through diapause, which sheds light on developmental plasticity in response to adverse conditions
- Not only do organisms, embryos, and stem cells undergo diapause/quiescence under adverse conditions, but cancer cells also can use the diapause-like mechanism to survive chemotherapy
- An in-depth understanding of the molecular and cell biological underpinning of the diapause will help to extend healthspan and reproductive lifespan

## Open Access

Open access for this article was enabled by the participation of University of California in an all-inclusive *Read & Publish* agreement with Portland Press and the Biochemical Society under a transformative agreement with UC.

## Abbreviations

AstC: Allatostatin-C
DH: diapause hormone
ESC: embryonic stem cells
FOX: forkhead box family of transcription factors
JH: juvenile hormone
LIF: leukemia inhibitory factor
Mmp: matrix metalloproteinases
NE: norepinephrine
NPC1L1: Niemann-pick C1 like 1
OA: octopamine
ROS: reactive oxygen species
TOR: target of rapamycin
